# Recombinase-independent AAV for anterograde transsynaptic tracing

**DOI:** 10.1186/s13041-023-01053-7

**Published:** 2023-09-15

**Authors:** Islam Faress, Valentina Khalil, Haruka Yamamoto, Szilard Sajgo, Keisuke Yonehara, Sadegh Nabavi

**Affiliations:** 1 Department of Molecular Biology and Genetics, Aarhus University, Aarhus, Denmark; 2 DANDRITE, The Danish Research Institute of Translational Neuroscience, Aarhus, Denmark; 3 Center for Proteins in Memory-PROMEMO, Danish National Research Foundation, Aarhus, Denmark; 4 Department of Biomedicine, Aarhus University, Høegh-Guldbergs Gade 10, 8000 Aarhus, Denmark; 5 Department of Biosystems Science and Engineering, ETH Zurich, Basel, Switzerland; 6 Multiscale Sensory Structure Laboratory, National Institute of Genetics, Mishima, Japan; 7 Department of Genetics, The Graduate University for Advanced Studies (SOKENDAI), Mishima, Japan

**Keywords:** AAV, Transsynaptic, Neural-circuit, Viral-tracing, Neuroanatomy

## Abstract

Viral transsynaptic labeling has become indispensable for investigating the functional connectivity of neural circuits in the mammalian brain. Adeno-associated virus serotype 1 (AAV1) allows for anterograde transneuronal labeling and manipulation of postsynaptic neurons. However, it is limited to delivering an AAV1 expressing a recombinase which relies on using transgenic animals or genetic access to postsynaptic neurons. We reasoned that a strong expression level could overcome this limitation. To this end, we used a self-complementary AAV of serotype 1 (scAAV1) under a strong promoter (CAG). We demonstrated the anterograde transneuronal efficiency of scAAV1 by delivering a fluorescent marker in mouse retina-superior colliculus and thalamic-amygdala pathways in a recombinase-independent manner in the mouse brain. In addition to investigating neuronal connectivity, anterograde transsynaptic AAVs with a strong promoter may be suitable for functional mapping and imaging.

**Supplementary Information:**

The online version contains supplementary material available at 10.1186/s13041-023-01053-7.

## Introduction

Significant progress has been made in the fields of neurophysiology and neuroanatomy since the discovery of viral approaches to map out brain connectivity. The viral approach is a powerful tool to label interconnected brain regions, allowing cell-type-specific identification and manipulation. Retrograde mapping of the presynaptic inputs to a specific brain region is achievable via different viral tools that vary in their degree of specificity (axonal vs. transsynaptic transport) and efficiency [1, 2]. Anterograde tracing has remained largely limited to labeling axonal input to the downstream postsynaptic region. However, the recent discovery of the adeno-associated virus serotype 1 (AAV1) property to label postsynaptic neurons transsynaptically advanced the field. AAV1 expressing a recombinase has been used in various studies to label and manipulate postsynaptic neurons. The major limitation is that a recombinase-dependent transgene should be expressed in downstream targets, which is doable only via transgenic animals or, alternatively, via viral expression postsynaptically, which requires prior knowledge of the target [1, 3, 4]. This study aims to optimize a method allowing recombinase-independent anterograde transsynaptic labeling of postsynaptic neurons in the mammalian brain.

## Results

We optimized the expression parameters to achieve stronger expression of an AAV1 vector that carries a green fluorescent protein (GFP). The vector is self-complementary, I.e., carrying double-stranded DNA and under short-CAG as a promoter at a high titer (>1.0 × 10E13 vg/ml) [5]. To test the efficiency of the vector, we conducted two separate experiments in well-characterized long-range monosynaptic and non-reciprocal visual and auditory pathways. In the first experiment, we injected the vector into the mouse retina and imaged the downstream target in the superior colliculus (SC) [6, 7]. In the second experiment, the virus was injected into the medial geniculate nucleus (MGn), and we imaged the basolateral amygdala (BLA) [8–11]. We also evaluated whether an AAV1 vector with an alternative (and possibly weaker) promoter, human Synapsin (hSyn), would yield a comparable level of transsynaptic anterograde labeling.

**Fig. 1:**
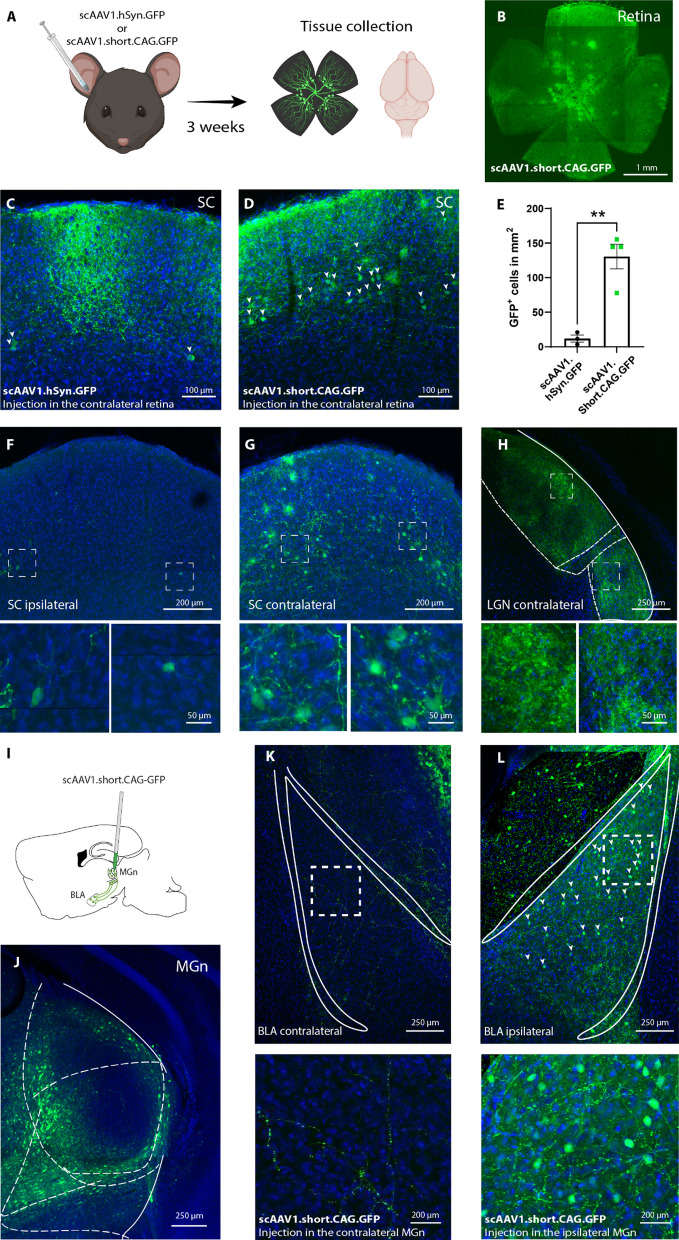
**A** Diagram showing the experimental timeline for the retinal injection of either scAAV1.hSyn.GFP (n = 3) or scAAV1.short.CAG.GFP (n = 4). **B** Image of a retina injected with scAAV1.short.CAG.GFP. **C**, **D** Representative image showing GFP-labeled neurons in the SC after scAAV1.hSyn.GFP (**C**) and scAAV.1 short.CAG.GFP (**D**) retinal injection; arrows point to labelled neurons. **E** The number of GFP-positive neurons in the SC is significantly higher in mice with scAAV1.short.CAG.GFP retinal injection (n = 4) compared to mice with scAAV1.hSyn.GFP retinal injection (n = 3; Unpareid t-test, p = 0.0027). **F–H** Representative images from the ipsilateral SC (**F**), contralateral SC (**G**), and LGN (**H**). The insets below are high-magnification images of the boxed regions. **I** Diagram showing the injection of AAV.1 short.CAG.GFP in the MGn and the anterograde transsynaptically labeled neurons in the BLA (n = 4). **J** Representative image of the injection site in the MGn. **K**, **L** Representative images showing that the ipsilateral BLA has GFP-positive neurons; arrows point to labelled neurons (**L**) after AAV1.short.CAG.GFP in the MGn, while the contralateral BLA (**K**) does not. The boxes below are high magnification of the respective regions. Results are reported as mean ± SEM. **p < 0.01

We delivered the scAAV1 GFP under either short.CAG or hSyn to the retina, and 3 weeks later, we harvested the brains and retinae to examine the GFP labeling postsynaptically (Fig. 1A–H). The two vectors were delivered separately at an equal titer and volume. We observed that scAAV1.short.CAG.GFP resulted in a significantly higher number of transsynaptically labeled GFP neurons in the SC, in comparison to scAAV1.hSyn.GFP (Fig. 1C–E). In the mice injected with scAAV1.short.CAG.GFP in the retina, we observed that the contralateral SC had robust neuronal GFP labeling with clear neuronal morphology. In contrast, the ipsilateral had very sparse labeling, which is consistent with basic retinal-collicular anatomy (Fig. 1F, G). Unlike the efficient transsynaptic anterograde labeling we observed in the SC from the retina, we did not observe any somatic labeling in the lateral geniculate nucleus (LGN). This lower labeling efficiency in the LGN compared to the SC is consistent with the original study where AAV1 has been reported to show anterograde transsynaptic labeling properties [3]. Next, we tested the intracerebral efficiency by injecting the scAAV1.short.CAG.GFP in the MGn. This resulted in robust GFP labeling only in the ipsilateral BLA with clear neuronal morphology. However, there was no labeling in the contralateral BLA, which is consistent with the known neuroanatomy (Fig. 1I–L) [8, 9]. To further confirm the directionality, from the MGn to the BLA, we injected a retro AAV2 vector expressing mRuby in the MGn and imaged downstream and upstream regions of the MGn. We did not observe any neural labelling in the BLA, as a downstream target. However, we observed numerous labelling in an MGn-upstream region cortical, the auditory cortex, consistent with the reported corticothalamic circuitry [11, 12] (Additional file 1: Fig. S1A–D). To confirm the neuronal specificity, we co-stained for the astrocytic marker, glial fibrillary acidic protein, GFAP, in the BLA. We did not observe any co-labeling of the transneuronally labelled GFP cells and the GFAP positive cells (Additional file 1: Fig. S1E).

## Discussion

Here we investigated the possibility of achieving anterograde transsynaptic labeling in a recombinase independent manner using an AAV1 vector. We demonstrated that scAAV1.short.CAG. GFP could transneuronally label downstream postsynaptic targets with a single virus injection in wild-type mice. After injecting the vector into the retina and the MGn, we observed reliable labeling in the SC and the BLA, respectively. Single-stranded AAV1 CAG GFP was reported to lack transneuronal anterograde labeling capacity, which might be due to the low expression level [3]. Consistent with this hypothesis, we observed that scAAv1.short.CAG.GFP yields more robust transsynaptic labeling in comparison to a possibly weaker promoter like scAAV1.hSyn.GFP. Our results indicate that the transsynaptic transfer of transgenes that express optogenetic/chemogenetic actuators might be achievable by using the AAV1 expressing the transgene of interest in the mammalian brain. Similarly, in an avian model, avian-adenoassociated virus (an AAV variant) shows recombinase-independent transneuronal labeling, putatively with a similar underlying mechanism to the AAV1 [13]. These viral tools will expand the potential utility of viral approaches for circuit interrogation in a pathway and cell-type specific manner. Future studies will be needed to optimize AAV-mediated transsynaptic tracing to allow for strict anterograde labeling and, thereby, make it applicable in reciprocally connected brain regions. Similarly, optimizations that allow for using AAV1 at a lower titer while maintaining the anterograde transsynaptic property might reduce the toxicity at the injection site.

### Supplementary Information

**Additional file 1**: **Figure S1 A **Diagram showing the injection of Retro/AAV.2hSyn.mRuby in the MGn. **B **Representative image of the injection site in the MGn. **C **Representative images showing DAPI nuclear staining (left), and the lack of retrograde labeling and dense axonal labeling (middle) in the BLA. The right panel shows the merge between the nuclear staining and the mRuby axonal labeling. The zoomed-in image shows the lack of overlap between the nuclear staining and the mRuby labeling. **D **Representative images of DAPI nuclear labeling (left) and axonal and retrograd neuronal mRuby expression in the auditory cortex (Actx) from the same mouse shown in panels B and C. The right panel shows a zoomed-in image of the merge of the nuclear staining and the mRuby labeling. **E **Representative images of DAPI nuclear labeling (left) and anterograde transsynaptically GFP labeled neurons (middle). Glial fibrillary acidic protein (GFAP) expressing cells in magenta and a merge between the previous panels on the three images of an ipsilateral amygdala from a mouse injected with scAAV1.short.CAG.GFP in the MGn (n=4). The images show the lack of colocalization between the transsynaptically labeled GFP^+^ neurons and the GFAP^+^ cells in the amygdala.

## Data Availability

The datasets used and/or analysed during the current study are available from the corresponding author upon reasonable request.
