# Supplementary material for: Recombinase-independent AAV for anterograde transsynaptic tracing
Source: Mol Brain. 2023 Sep 15;16:66. doi: 10.1186/s13041-023-01053-7 (PMC10504749; doi:10.1186/s13041-023-01053-7)
Supplement: Supplementary file 1 — Additional file 1: Figure S1 A Diagram showing the injection of Retro/AAV.2hSyn.mRuby in the MGn. B Representative image of the injection site in the MGn. C Representative images showing DAPI nuclear staining (left), and the lack of retrograde labeling and dense axonal labeling (middle) in the BLA. The right panel shows the merge between the nuclear staining and the mRuby axonal labeling. The zoomed-in image shows the lack of overlap between the nuclear staining and the mRuby labeling. D Representative images of DAPI nuclear labeling (left) and axonal and retrograd neuronal mRuby expression in the auditory cortex (Actx) from the same mouse shown in panels B and C. The right panel shows a zoomed-in image of the merge of the nuclear staining and the mRuby labeling. E Representative images of DAPI nuclear labeling (left) and anterograde transsynaptically GFP labeled neurons (middle). Glial fibrillary acidic protein (GFAP) expressing cells in magenta and a merge between the previous panels on the three images of an ipsilateral amygdala from a mouse injected with scAAV1.short.CAG.GFP in the MGn (n=4). The images show the lack of colocalization between the transsynaptically labeled GFP+ neurons and the GFAP+ cells in the amygdala. [file 13041_2023_1053_MOESM1_ESM.docx]

**Recombinase-Independent AAV for Anterograde Transsynaptic Tracing**

Islam Faress^1,2,3,4,^**^*^**^,^
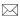
 , Valentina Khalil^1,2,3,^**^*^**, Haruka Yamamoto^2,4^**^*^**, Szilard Sajgo^5^, Keisuke Yonehara^2,4,6,7^,

Sadegh Nabavi1,2,3,

Department of Molecular Biology and Genetics, Aarhus University, Aarhus, Denmark

1. DANDRITE, The Danish Research Institute of Translational Neuroscience
2. Center for Proteins in Memory – PROMEMO, Danish National Research Foundation
3. Department of Biomedicine, Aarhus University, Aarhus, Denmark
4. Department of Biosystems Science and Engineering, ETH Zurich, Basel, Switzerland.
5. Multiscale Sensory Structure Laboratory, National Institute of Genetics, Mishima, Japan
6. Department of Genetics, The Graduate University for Advanced Studies (SOKENDAI), Mishima, Japan

*These authors contributed equally

- Corresponding author
- ***Correspondence**: [Islam.faress@Dandrite.au.dk](mailto:Islam.faress@Dandrite.au.dk)

Department of Biomedicine at Aarhus University, Høegh-Guldbergs Gade 10, 8000 Aarhus https://orcid.org/0009-0000-2218-9180

**Keywords:** AAV, Transsynaptic, Neural-circuit, Viral-tracing, Neuroanatomy

**This file includes:**

**Supplementary figure S1**

**Material and Methods**

**
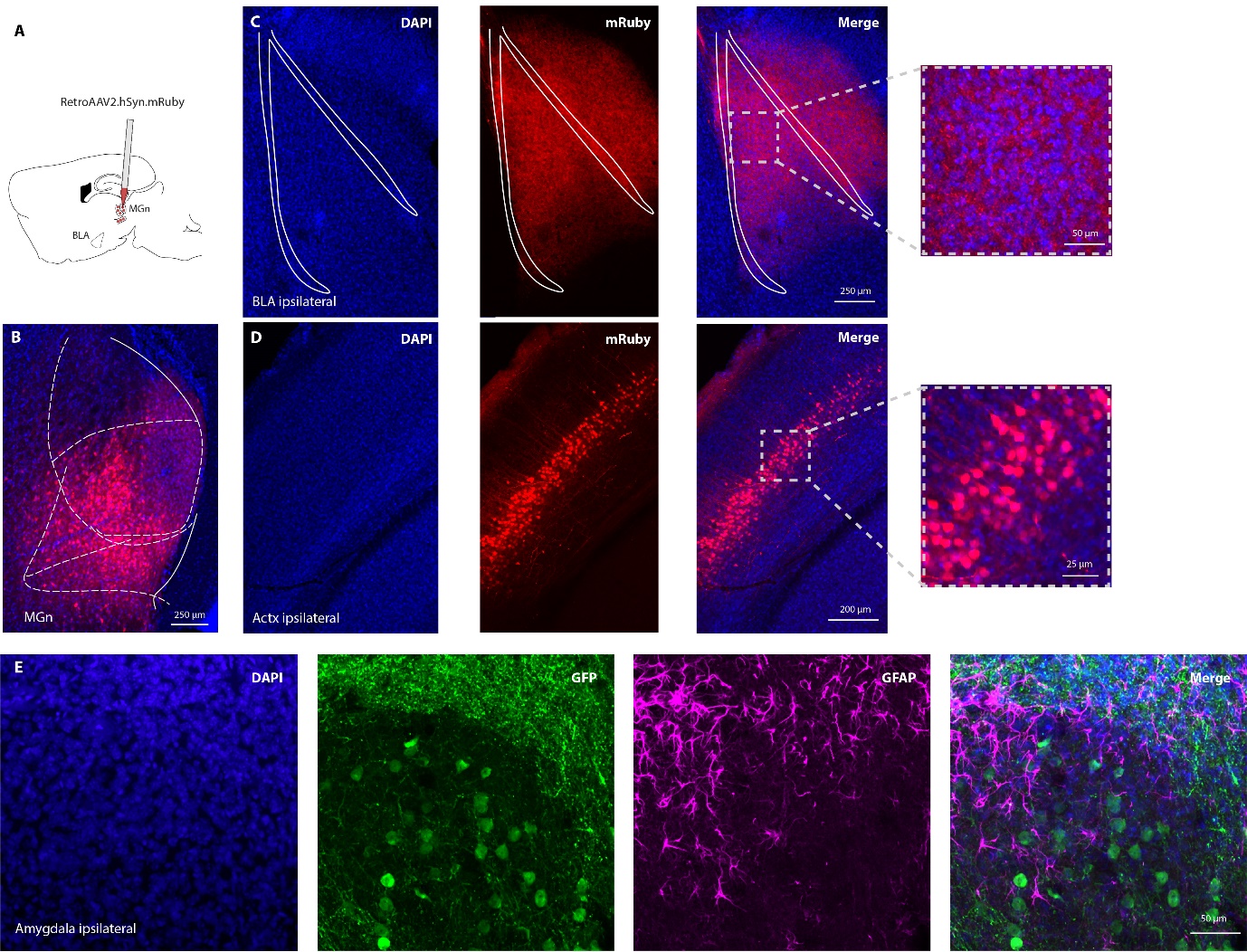
**

**Figure S1 A)** Diagram showing the injection of Retro/AAV.2hSyn.mRuby in the MGn. **B)** Representative image of the injection site in the MGn. **C)** Representative images showing DAPI nuclear staining (left), and the lack of retrograde labeling and dense axonal labeling (middle) in the BLA. The right panel shows the merge between the nuclear staining and the mRuby axonal labeling. The zoomed-in image shows the lack of overlap between the nuclear staining and the mRuby labeling. **D)** Representative images of DAPI nuclear labeling (left) and axonal and retrograd neuronal mRuby expression in the auditory cortex (Actx) from the same mouse shown in panels B and C. The right panel shows a zoomed-in image of the merge of the nuclear staining and the mRuby labeling. **E)** Representative images of DAPI nuclear labeling (left) and anterograde transsynaptically GFP labeled neurons (middle). Glial fibrillary acidic protein (GFAP) expressing cells in magenta and a merge between the previous panels on the three images of an ipsilateral amygdala from a mouse injected with scAAV1.short.CAG.GFP in the MGn (n=4). The images show the lack of colocalization between the transsynaptically labeled GFP^+^ neurons and the GFAP^+^ cells in the amygdala.

**Materials and methods**

Retinal and brain injections

For intravitreal viral injections, mice were anesthetized with an intraperitoneal injection of fentanyl (0.05 mg/kg body weight; Actavis), midazolam (5.0 mg/kg body weight; Dormicum, Roche), and medetomidine (0.5 mg/kg body weight; Domitor, Orion) mixture dissolved in saline. We made a small hole at the border between the sclera and the cornea with a 30-gauge needle. 1.5 μl of the AAV vector was injected into the vitreous of the eye using a blunt-end microsyringe (Ito Corporation, #MS*E05). Mice were returned to their home cage after anesthesia was reversed by an intraperitoneal injection of flumazenil (0.5 mg/kg body weight; Anexate, Roche) and atipamezole (2.5 mg/kg body weight; Antisedan, Orion Pharma) mixture dissolved in saline. AAV delivery to MGn (0.3 μl), GFP (Invitrogen, CAB4211, 1:1000; amplified with Alexa 488, Invitrogen, A-11011, 1:1000)  and GFAP (Abcam, ab116010, 1:1000; amplified with Alexa 647, Invitrogen, A-31573, 1:1000) immunohistochemical amplification and GFP quantification were done as described in (Khalil et al., 2023).

Confocal imaging

Imaging was performed using the ZEN software (ZEN 2.5, blue edition) and a ZEISS confocal microscope (LSM 780/Axio Imager 2). The images were taken as z-stack images (z-step size = 4 μm) in the respective areas using 10× (EC Plan-Neofluar 10x/0.30) or 20x (EC Plan-Neofluar 20x/0.50 M27) objectives. Alexa 488 was excited at 488 nm and detected through a bandpass filter of 491–535 nm, while the DAPI was stimulated at 405 nm and detected through a 410–483 nm bandpass filter.   Alexa 647 was excited at 633 and detected through a bandpass filter of 638-755 nm. mRuby was excited at 561 nm and detected through a bandpass filter of 561–640 nm.

Statistics

Statistical analyses were performed by using GraphPad Prism 9. All the data are represented as mean ± SEM, and they were tested for normality using Shapiro-Wilk. If the data represented a normal distribution, a parametric test was used. The statistical methods and the corresponding p-values are reported in the figure legend.
